# Nearly one-quarter of patients say mechanical heart valve disturbs sleep

**Published:** 2017

## Introduction

‘For some patients, the closing sound of their mechanical heart valve reduces their quality of life, disturbs their sleep, causes them to avoid social situations, and leads to depression and anxiety’, said lead author Dr Kjersti Oterhals, a nurse researcher at Haukeland University Hospital in Bergen, Norway. He was speaking at EuroHeartCare 2017 in Sweden.

This study investigated how the noise of a mechanical heart valve affected patients’ lives, in particular their sleep, and whether there were any differences between women and men.

In April 2013 all 1 045 patients who had undergone aortic valve replacement at Haukeland University Hospital between 2000 and 2011 were invited to participate in a postal survey. Of the 908 patients who responded, 245 had received a mechanical valve and were included in the current analysis.

Patients were asked if the valve sound was audible to them or others, if they sometimes felt uneasy about the sound, if the sound disturbed them during daytime or during sleep, and whether they wanted to replace the mechanical valve with a soundless prosthetic valve if possible. Patients ranked the noise on a scale of 0 (does not disturb them at all) to 10 (causes maximum stress). The Minimal Insomnia Symptom Scale, which consists of three questions about sleep, was used to give patients a score of 0 to 12 for insomnia.

Patients were 60 years old on average and 76% were men. Nearly one-quarter (23%) said the valve sound disturbed them during sleep and 9% said it disturbed them during the day. Some 28% wanted to replace their valve with a soundless prosthetic valve if possible. Over half (51%) said the noise was often or sometimes audible to others, but only 16% said they sometimes felt uneasy about others hearing it.

The researchers found that 87% of men and 75% of women said that they were able to hear the closing sound of their mechanical valve. Women were more disturbed by the valve sound than men.

Some 53% of the respondents had no insomnia, 31% had subclinical insomnia, and 17% had moderate to severe insomnia. Valve noise perception was the strongest predictor of insomnia, followed by age and female gender. There was a linear association between insomnia and valve noise perception, and the more patients considered the valve noise a disturbance in daily life, the more insomnia they reported.

Dr Oterhals said ‘Almost one-fourth of patients said that the sound of their mechanical heart valve makes it difficult for them to sleep. Most of us need a quiet environment when we are going to sleep and these patients found it hard to ignore the noise from the valve.’

Not all patients are aware before surgery that they may hear their mechanical valve, and while most get used to it, for some it is troublesome for many years. ‘One female patient said to me, “I will never have silence around me again” when she realised she would hear the noise 24 hours a day for the rest of her life’, said Dr Oterhals.

The most common ways patients coped with the noise when trying to sleep were to sleep on their right side, which reduced the valve noise, put the duvet around their bodies to isolate the sound, listen to music and do relaxation exercises. Ear plugs were not effective and made the valve noise louder.

Dr Oterhals said: ‘We are not very proactive about this issue at the moment. It would improve many patients’ quality of life if we asked them about valve noise and provided advice to those who find it distressing.’

